# Agonistic interactions between the honeybee (*Apis mellifera ligustica*) and the European wasp (*Vespula germanica*) reveal context-dependent defense strategies

**DOI:** 10.1371/journal.pone.0180278

**Published:** 2017-07-05

**Authors:** Michelina Pusceddu, Ignazio Floris, Franco Buffa, Emanuele Salaris, Alberto Satta

**Affiliations:** Dipartimento di Agraria, sezione di Patologia vegetale ed Entomologia, Università di Sassari, Sassari, Italy; University of California San Diego, UNITED STATES

## Abstract

Predator–prey relationships between sympatric species allow the evolution of defense behaviors, such as honeybee colonies defending their nests against predatory wasps. We investigated the predator–prey relationship between the honeybee (*Apis mellifera ligustica*) and the European wasp (*Vespula germanica*) by evaluating the effectiveness of attack and defense behaviors, which have coevolved in these sympatric species, as well as the actual damage and disturbance caused to the colonies under attack. Attack and defense behaviors were recorded in front of the hive to observe attacks at the hive entrance (68 attacks in 279 h) and at ground level on isolated and weakened honeybees close to the hive (465 attacks in 32 h). We found that *V*. *germanica* attacked the hive entrance infrequently due to the low success rate of this strategy and instead preferred a specialized attack method targeting adult honeybees at ground level, demonstrating opportunistic scavenger behavior. Individual honeybees usually responded effectively to an attack by recruiting an average of two nestmates, causing the wasp to flee, whereas collective balling behavior was only observed on four occasions. *V*. *germanica* does not appear to disrupt the foraging activity of the colonies under attack. We found that agonistic events supported by other nestmates were typically the most intense ones, involving physical combat and prolonged attacks at the entrance to the hive. These observations support the hypothesis that *A*. *mellifera ligustica* can adapt its behavior to match the severity of the threat and the context of the attack.

## Introduction

Cooperation among individuals is observed in many phylogenetically diverse taxa and has facilitated the evolution of sociality in the animal kingdom [1,2]. The main advantages of sociality, promoted by natural selection, include more efficient vigilance against predators, a better ability to identify food sources, and the greater survival of developing brood. However, life within a group also presents certain disadvantages, one of the most significant being the ease with which predators can detect prey. For this reason, nest protection to reduce vulnerability is another central aspect in the evolution of sociality [3,4].

Eusocial insects such as the honeybee (*Apis mellifera*) adopt numerous general and behavioral defense mechanisms against their predators. General mechanisms include nest architecture, site and visibility, as well as species-dependent morphological adaptations such as the size of an individual [5]. In contrast, behavioral defenses are specific to particular enemies and require the prior identification of the predator based on olfactory, visual or tactile cues, recognition of movement, and information from previous encounters [6,7,8]. Behavioral defense can also depend on agonistic behavior by the invader during an encounter, and in eusocial insects, on the caste to which the occupant and/or intruder belong [9]. In the latter case, individuating and blocking specific predators in honeybee societies is the responsibility of guard bees. These bees adopt specialized behaviors that dissuade attacks by invertebrate predators and conspecifics from other colonies, thus preventing the loss of food and brood, and they also recruit “soldiers” to defend the nest against more aggressive predators [10,11]. Defense behavior in the Asiatic honeybee (*Apis cerana*) was recently shown to vary not only according to the predator, but also based on the context in which the attack takes place, e.g. minimal peril caused by an attack on a single forager contrasting with the substantial threat caused by an attack at the nest entrance [12].

Wasps are major invertebrate enemies of honeybees, invading hives to steal honey, pollen, larvae and adults to provide sugar and protein for themselves and their offspring [13,14]. The defense mechanisms used by *A*. *mellifera* against the Asian predatory hornet (*Vespa velutina*) has been studied in detail due to the predatory success of the wasp [15] and the damage caused by its introduction into Europe [16]. However, the study of relationships between sympatric species is also necessary even though the predators are less dangerous [17] because such relationships may offer insight into the evolution of defense behaviors [18,19]. We therefore investigated the predator–prey relationship between two sympatric species of social Hymenoptera, namely the honeybee (*Apis mellifera ligustica*) and the European wasp (*Vespula germanica*) also known as the German wasp or German yellowjacket, in a representative area of the European Mediterranean region (Sardinia, Italy). We evaluated the effectiveness of behavioral displays of attack and defense which have co-evolved in these two species, the defense mechanisms in various peril contexts, and the potential damage and disturbance caused by this predator to the honeybee colony under attack.

## Materials and methods

### Experimental apiary

The experimental apiary was set up in the northwest of Sardinia in March 2014, inside the experimental farm of the University of Sassari Department of Agriculture (latitude 40°46'23'', longitude 8°29'34''). The apiary comprised 18 *A*. *mellifera ligustica* colonies maintained in new Dadan-Blatt hives containing 10 combs each. They were checked every week to confirm the presence of the queen, as well as pollen and nectar provisions. We also monitored the sanitary status for evidence of microbial infections and varroosis [20].

### Behavioral observations

Agonistic events between *V*. *germanica* and *A*. *mellifera ligustica* were examined in two different contexts, one at the hive entrance (which was regularly patrolled by guard bees) and the other on the ground close to the hive, where weakened and dead bees were present. The behavioral observations were based on the “all occurrences sampling” method [21] in which we recorded the frequencies of a series of behavioral events as set out in the ethogram described below.

*Attacks* at the nest entrance were recorded in 2014 and 2015 throughout September and October, when the predatory activity of wasps is more intense due to their higher nutritional requirements during reproduction and rearing of offspring [22]. Each colony was recorded for two 15-min sessions per day using a Canon LEGRIA HF R506 video camera placed ~20 cm from the opening of the hives. Recordings were taken during the hottest part of the day (between 9:30 am and 15:30 pm) when the wasps were most active. A total of 279 h of video footage was recorded (63 h in 2014 and 216 h in 2015) and all 18 colonies were observed for the same duration (15.5 h). Subsequently, two operators independently screened the video recordings using a slow motion system (VLC software v2.2.0) and the agonistic behaviors observed were used to establish an ethogram as described below. The ethogram was supplemented with further “attack” and “defense” behaviors not observed by us but reported in the literature for similar species, or in these two species facing different antagonists. This approach allowed us to evaluate the repertoire of agonistic behavior between *V*. *germanica* and *A*. *mellifera ligustica* in a wider context. The frequency (number of events per unit of time) was reported for all the recorded attack and defense behaviors.

*Attacks* at ground level (only on individuals still alive and close to the hive) were monitored in 2015 on the same colonies, concurrently with some of the observations at the nest entrance. These observations were conducted by sight, without using the video camera, for a total of 32 h. Two operators simultaneously observed the ground surface under three hives in two sessions per day, each lasting 10 min. The frequency (number of events per unit of time) was reported for all the observed attack and defense behaviors.

### Effect of predator attacks on bee foraging activity

The 15-min video clips taken at the nest entrance in each colony were used to evaluate the disturbance caused by wasps on the foraging activity of the honeybees. We compared the frequency of pollen foragers entering the hive 5 min after wasp *attack* (“attack” context) with the frequency at random times before the *attack* (“control” context) over a fixed 2-min interval. The comparisons were carried out for 27 agonistic events observed in 2015 to account for any interference that prevented us counting the number of pollen foragers, e.g. continuation of *balling*, successive *attacks*, or other bees blocking the view of the video camera.

### Agonistic support

To determine whether there was a correlation between the degree of *agonistic support* and the intensity of predator aggression, all *attacks* at the nest entrance were divided into two behavioral categories described as *threats* (attacks in which the defender did not make physical contact with the predator) and *fights* (where physical contact was involved) [23,24]. For each agonistic event, we recorded the duration of the *attack*, the number of supporters intervening to help a nestmate under attack (in the case of individual support) and any observed cases of *balling*.

### Ethogram

#### Wasp attack behaviors observed in this study

***Attack***—The wasp swoops down to the landing board or to ground level below the entrance plate, grasps the bee from above with its forelegs and starts biting it (usually between the caput and thorax).

***Fight***—The predator and prey are involved in a physical encounter which may include instances of biting, aggressive gripping, and spinning on a surface or in flight (S1 Photo).

***Entering the hive***—A wasp may be able to enter the nest if it is overlooked by the bees. Wasps may also enter the nest after contact with bees, i.e. following *antennation* or following a *fight*.

***Predation***—The wasp kills the honeybee. The wasp usually goes on to dismember and consume the honeybee or to carry off parts to its offspring (see below). In some cases, the wasp may also eat the contents of the honey stomach [13].

***Sequestration***—After *predation*, and having divided the honeybee into three parts (caput, thorax and abdomen), the wasp flies off with one of them, usually the thorax [25,26].

***Escape***—The wasp *escapes* when the *attack* has not been successful and one or more honeybees defend themselves or their nest effectively (S1 Movie).

#### Wasp attack behaviors not observed in this study

***Coalition attack***—A coalition of several wasps launches an *attack*. This behavior has been reported for the Asian giant hornet (*Vespa mandarinia*), which has developed a strategy of group hunting: certain individuals pillage while others defend the site against conspecifics from other colonies [27].

#### Nest defense behaviors observed in this study

***Antennation or antennal boxing***—This is often the first physical contact between the occupant and invader, and most likely facilitates the recognition of intruders and conspecifics [28]. It is defined as asymmetric when a dominant and a submissive can clearly be distinguished from the behavioral display of the two opponents, or symmetric when such a distinction is not possible [29].

***Threat***—The typical behavior of a honeybee in the presence of a conspecific intruder or other predator, consisting of open mandibles and the adoption of the so-called C posture (gaster flexion with or without extension of the sting) [9].

***Agonistic support***—Altruistic behavior in which a honeybee helps another involved in a conflict, thus facing a potential risk. It may involve a single bee or several bees (supporters) that come to the aid of their nestmates.

***Balling***—The formation of a ball of bees around a wasp until the latter is killed or becomes harmless (S2 Photo). In *heat-balling* the wasp succumbs to the heat inside the ball because hornets and wasps have a lower thermal tolerance than bees [30,31]. In *asphyxia-balling*, the heat inside the ball can be lethal to the predator but it dies due to the increased concentration of CO_2_ in the hemolymph which causes asphyxiation [32,33].

***Killing and removal of the predator***—Wasps can be killed by a single bee sting or the stings of several bees, or by *balling* (see above). The dead or dying wasp is then removed from the nest or landing board.

#### Nest defense behaviors not observed in this study

***Bee carpet***—A large proportion of the colony regroups on the landing board and along the sides of the hive, forming a “*bee carpet*” [19]. This behavior in *A*. *mellifera ligustica* has been observed against the European hornet (*Vespa crabro*) [13].

***Shimmering or shaking signal***—When a wasp is seen, bees at the entrance of the hive simultaneously vibrate their abdomens for a few seconds, resulting in the emission of a loud *hissing* noise caused by the movement of their wings [19]. This behavior has been observed in the giant honeybee (*Apis dorsata*) [34,35], the dwarf honeybee (*Apis florea*) [5], *A*. *cerana* [36], *A*. *cerana nuluensis* [15] and *A*. *mellifera cypria* [37] against *V*. *velutina* and the oriental hornet (*Vespa orientalis*). *A*. *mellifera ligustica* also generates a hissing noise in the presence of *V*. *crabro* albeit without the *shimmering* behavior [18,13]. *Shimmering* is considered a visual signal for the predator and appears to have evolved in order to dissuade the latter from attacking, i.e. it is an honest alert signal that reduces the likelihood of predator success [7,34].

***Interruption of foraging***—The *interruption of foraging* activity in a colony under attack by *V*. *velutina* has been reported in *A*. *cerana* [38] and *A*. *mellifera cypria* [37].

***Retreat into the nest***—Complete *retreat into the nest* during an *attack* has been described in *A*. *cerana* [15] and *A*. *mellifera cypria* [37], as well as the Cape honeybee (*A*. *mellifera capensis*), the African honeybee (*A*. *mellifera scutellata*) and the Carniolan honey bee (*A*. *mellifera carnica*) [39].

***Bees attack***—Africanized bees are characterized by a higher propensity than European bees to switch from collective nest defense to attack behavior [40]. The response of Africanized bees is also faster, more aggressive, and involves the recruitment of more nestmates [18]. Attack behavior has also been observed in *A*. *mellifera cypria* towards *V*. *orientalis* [37].

### Statistical analysis

The disturbance of foraging activity was measured by comparing the number of pollen foragers in the attack context to the number of pollen foragers in the control context using the Wilcoxon signed rank test (paired comparisons). A chi-squared test was used to measure the proportional difference in support events (individual *agonistic support* and *balling*) between the *threat* and *fight* categories. To reduce the chance of a type I error, continuity correction was used for the chi-squared tests because the sample size was less than 200 [41].

The Wilcoxon rank sum test (unpaired comparisons) was used to compare the number of supporters in the *threat* and *fight* categories (excluding *balling*). We also tested for correlation (non-parametric Spearman correlation) between the number of supporters and the duration of *attacks*. To reduce the chance of a type I error in this analysis, we used Bonferroni correction in the case of multiple testing with significance set at α = 0.05/2 = 0.025. All tests were carried out using R v3.0.2 implemented with library (exactRankTests) and library (coin).

**Raw experimental data are available in supporting materials (**S1 Data File).

## Results

### Wasp attack

We observed 68 *attacks* at the hive entrance in 279 h of video footage, specifically 11 *attacks* in 2014 (63 h) and 57 in 2015 (216 h) representing ~0.24 *attacks* per hour. The most frequent outcome was wasp *escape* (55 events, 80.9%) and the least frequent was bee *predation* (1 event, 1.5%). On three occasions (4.4%) the wasp was observed *entering the hive* and coming out alive. On another three occasions it was not possible to confirm the fate of the wasp because the observation session terminated while the wasp was still inside the hive. The average *attack* time was 3.5 ± 0.4 s.

We observed 465 *attacks* at ground level in 32 h, and these only targeted isolated bees (~14.5 *attacks* per hour). In this case, the outcome was more balanced. Bee *predation* was observed 226 times (48.6%) and in 91 of these cases *sequestration* also occurred. The wasp was chased away 239 times (51.4%), which is a much lower proportion compared to hive entrance *attacks*. The attack behavioral display data are summarized in Table 1.

**Table 1:** Attack behavioral display by *V*. *germanica* against colonies of *A*. *mellifera ligustica*.

ATTACK BEHAVIORS	HIVE ENTRANCE (68 *attacks* in 279 h)		ON THE GROUND (465 *attacks* in 32 h)	
	n	%	n	%
Antennation*	11	16.2	-	-
*Predation*	1	1.5	226	48.6
Sequestration*	-	-	91	19.6
*Entering the hive*	6	8.8	-	-
*Escape*	55	80.9	239	51.4

* At the hive entrance, *antennation* may occur in isolation or in addition to other behaviors so the total number of events is greater than the number of *attacks*. On the ground, *sequestration* occurs in addition to *predation* in a subset of *predation* events so the total number of events is again greater than the number of *attacks*.

### Nest defense

We did not observe a collective attack against any of the colonies so our data only represent defense behaviors against individual wasps. Among the 68 agonistic events observed at the hive entrance, 28 cases (41.2%) involved a single bee defending itself or the nest successfully, thus causing the wasp to flee (S2 Movie). In the remaining 40 *attacks* (58.8%), other bees from the same nest came to the rescue. *Agonistic support* occurred in 90% of cases, involving an average of 1.9 ± 0.2 supporters per *attack* (S3 Movie). *Balling* was observed in 10% of cases. In six cases (8.8%), the wasp was killed and removed from the landing board. *Agonistic support* of the bees under attack was never observed among the 465 ground level *attacks* close to the hive. The defense behavioral display data are summarized in Table 2.

**Table 2:** Defense behavioral display by *A*. *mellifera ligustica* attacked by *V*. *germanica* when *predation* was not observed.

DEFENSEBEHAVIORS	HIVE ENTRANCE(68 *attacks* in 279 h)		ON THE GROUND (465 *attacks* in 32 h)	
	n	%	n	%
*Single bees*	28	41.2	465	100
*Agonistic support*	36	90	-	-
*Balling*	4	10	-	-
Killing of the wasp*	6	8.8	-	-

* This outcome is additional to the other behaviors so the total number of events is greater than the number of *attacks*.

### Disturbance of foraging

We did not observe any disturbance of foraging activity when the colony was under attack. Indeed, there were no statistically significant differences between the frequency of foraging in the attack context (24.2 ± 4.1) and in the control context (23.0 ± 3.4) in 2015 (*U* = 154, *N1 = N2* = 27, *P* = 0.6378).

### Agonistic support

**Fig 1:**
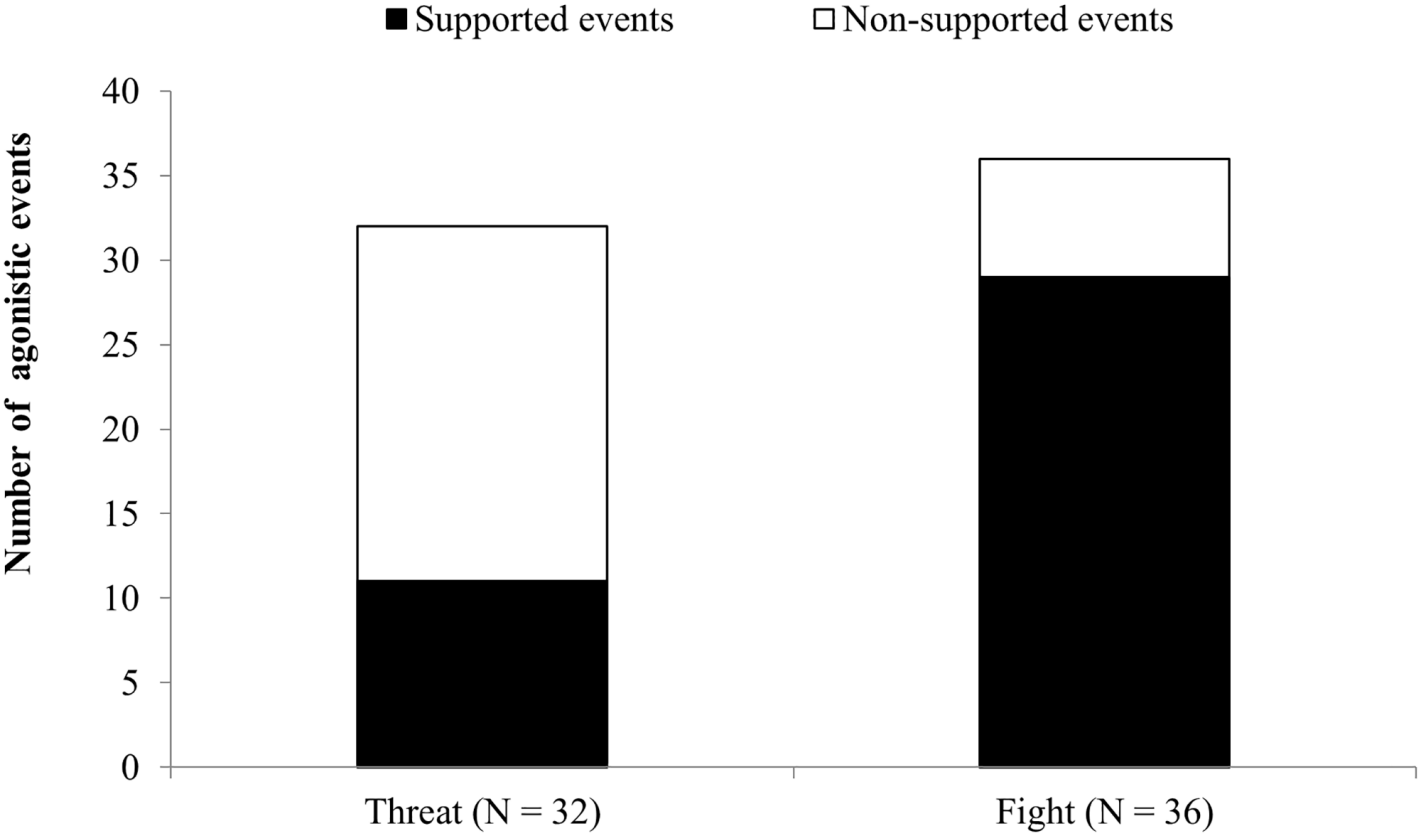
Number of supported and unsupported events classed as *threats* (agonistic interaction without physical contact) and *fights* (agonistic interaction with physical contact). The difference between the two groups was highly significant (chi-squared test, *P* < 0.001). N = number of agonistic events observed in 18 colonies.

**Fig 2:**
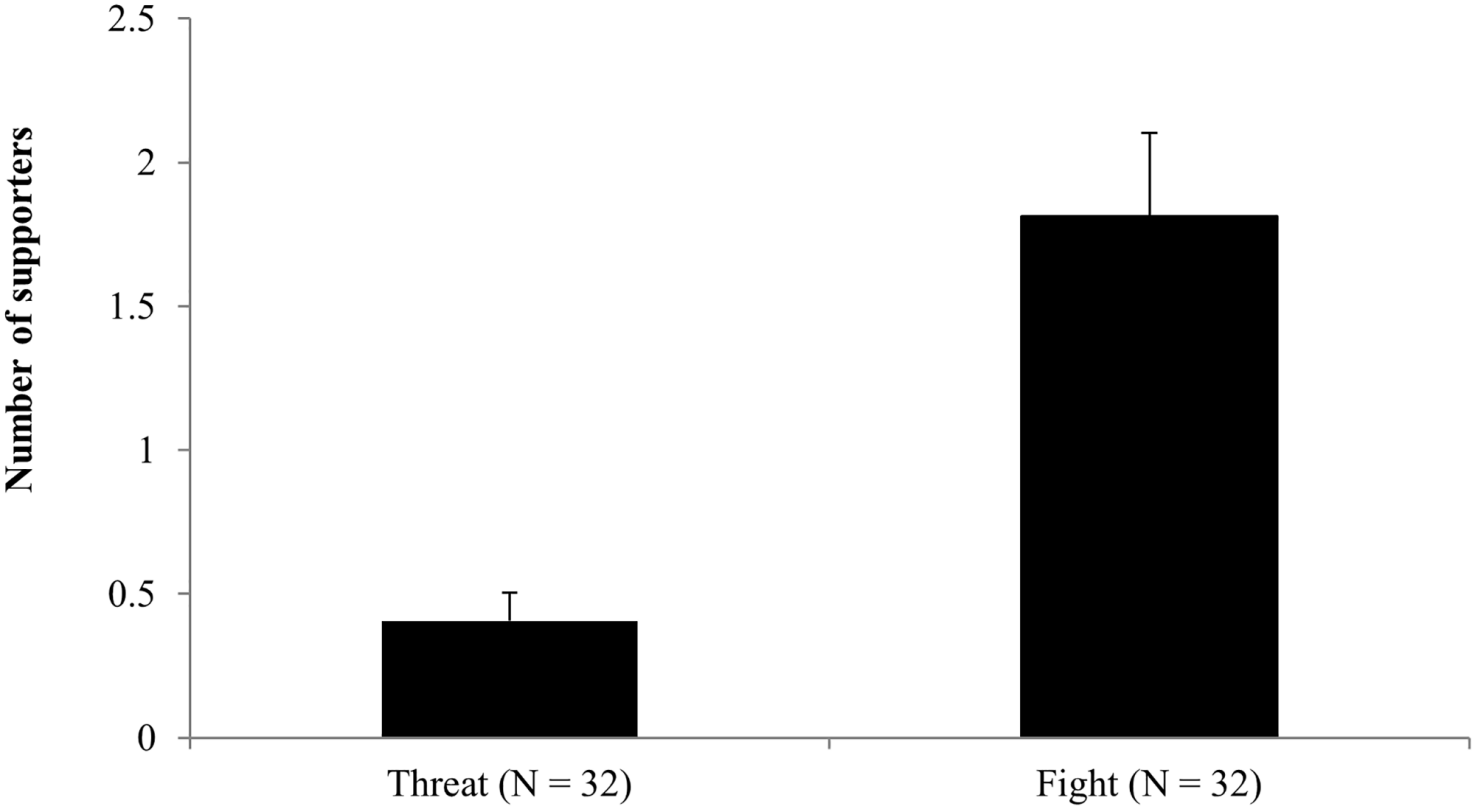
Number of supporters (mean ± standard error) for the agonistic events classed as *threats* (agonistic interaction without physical contact) and *fights* (agonistic interaction with physical contact). The 68 agonistic events we observed included four cases of *balling* which are excluded from the analysis. The difference between the two groups was highly significant (Wilcoxon rank sum test, unpaired comparisons *P* < 0.001). N = number of agonistic events observed in 18 colonies.

The agonistic events most commonly supported by nestmates either individually or by *balling* were those involving physical contact (*fights*) rather than warning behavior (*threats*). Accordingly, we observed a statistically significant difference between the number of supported *threats* and the number of supported *fights* as shown in Fig 1 (*chi-squared* = 13.07, *df* = 1, *P* < 0.001). Moreover, when *balling* events were excluded, the average number of supporters was significantly higher in *fights* than *threats*, as shown in Fig 2 (*U* = 221, *N1 = N2* = 32, *P* < 0.001). There was also a positive correlation between the number of supporters and the duration of *attack* (*S* = 20653, *P* < 0.001, *rho* = 0.53). *Agonistic support* was observed only at the hive entrance, not at ground level.

## Discussion and conclusions

Our data revealed that *V*. *germanica attacks* on nest of *A*. *mellifera ligustica* occur infrequently, particularly *attacks* on the landing board of the hive, so there is a low risk of predation. Only in extremely rare cases did the predator manage to overcome the barrier of guard bees, enter the hive, pillage it and *escape*. Instead, *V*. *germanica* predatory activity is clearly directed at ground level, targeting weak or isolated bees that have fallen from the entrance plate. This is a specialized form of attack, as opposed to a direct attack on the hive, which achieves high predation efficiency. It can be explained by the optimal foraging theory, which postulates a trade-off between energy returns and mortality due to predation [26,42,43,44]. Our observations revealed a compromise between the reward obtained and the risk taken by the wasp, with a direct *attack* on the hive entrance attracting a greater risk than *attacks* on isolated bees. Similar observations have been reported in other species: for example, the isolation of foragers is essential to improve the hunting efficiency of hornets in the vicinity of *A*. *dorsata* nests because this species is extremely effective in repelling hornets by *shimmering* [34].

Our observations also indicated that *V*. *germanica* is predominantly a solitary predator, because we found no evidence of coordinated attacks involving other conspecifics. In contrast, competition for food and pillaging among wasps were observed during *predation*. This probably reflects the individual and independent foraging typology of this species [44]: individuals from different colonies can find themselves at the same foraging site, explaining why each individual defends its own prey [26]. In contrast to predators such as *V*. *velutina*, *V*. *crabro* [45], and *V*. *tropica* [34], *V*. *germanica* has never been observed attacking forager bees in flight and returning to the hive, only bees on the ground or on the landing board.

The relatively weak *predation* practiced by *V*. *germanica* is confirmed by the ability of 1–3 bees to repel an *attack* without recourse to truly collective defense strategies such as *balling*. Indeed, *balling* by *A*. *mellifera ligustica* against *V*. *germanica* has never been reported before, and we observed this behavior only four times throughout our observation period. In contrast, *balling* is deployed much more frequently against Asian wasps [18,38]. This is important because *balling* often kills some of the participating bees in addition to the predator, suggesting that *A*. *mellifera ligustica* regulates its defense behavior depending on the intensity of the threat in order to prevent unnecessary sacrifices [7, 46]. We also saw no evidence of alternative collective defense strategies such as a *bee carpet* or *shimmering*, which are often deployed against *V*. *crabro* [13]. Again this suggests that *A*. *mellifera ligustica* adjusts its defense strategy in response to different predators, which can likewise be interpreted as a trade-off between the involvement of the colony in collective defense (with the associated risks discussed above) and the peril represented by the predator.

Our study also revealed that more intense agonistic events (i.e. *fights* rather than *threats*) attract stronger support from nestmates, and showed a correlation between the degree of support and the duration of *attack*. This suggests that *A*. *mellifera ligustica* can adapt its defense behavior according to the context of the peril [12]. The hypothesis is further supported by the different defense responses observed at the hive entrance and on the ground. Hive entrance *attacks* usually attracted supporters in the events because such *attacks* are obviously recognized as a potential form of peril for the entire colony. On the other hand, we observed that *attacks* against those individuals which had fallen from the hive plate were never supported. Possibly, these individuals were no longer recognized as nestmates.

The distinct nestmate-recruiting capacity observed in different behavioral peril categories probably reflects the emission of warning signals such as alarm pheromones by bees that are under attack [6,12]. In contrast to recent observations in colonies of *A*. *mellifera ligustica* attacked by *V*. *velutina* in Liguria, Italy (Cervo, personal communication), we never observed the *interruption of foraging* or complete *retreat into the nest*. We can therefore exclude the possibility that vibration stop signals are used to recruit supporters [12]. Indeed, we found no evidence that *V*. *germanica* disrupts *A*. *mellifera ligustica* foraging activity, providing more support for the hypothesis that the prey–predator relationship between these two sympatric species has reached a state of balance and that *V*. *germanica* need not be considered a threat to apiculture. However, to exclude the threat to bee foraging activity completely, further observations are required in areas with a greater density of wasp colonies.

Docile characteristics are preferred when selecting genetic lines of *A*. *mellifera* and several methods have therefore been developed to evaluate the aggression of reared honeybees. It follows that an understanding of agonistic behavioral displays in *A*. *mellifera* against natural enemies could be used to develop more effective tests to replace the current evaluation methods, which have been called into question [47]. Indeed, the method recommended by Apimondia (International Federation of Beekeepers’ Associations) is based on subjective evaluation by the operator on a four-point scale, where one point is assigned to the most aggressive bees and four points to the most docile, thus indicating the protective equipment the beekeeper must use [48]. This method does not account for climatic, chemical, visual, social and environmental variables that can play a role in the aggressive behavior of a colony, and is therefore too subjective and difficult to reproduce. A much more selective method has been developed to distinguish between aggressive and docile states in *A*. *mellifera* colonies representing the subspecies *carnica*, *scutellata* and *capensis* [39]. Our data could also facilitate the selection of genetic lines of honeybees that are less hostile to humans while maintaining aggressive behavior towards their natural enemies.

In conclusion, our study provides insight into the mechanisms of attack and defense deployed by *V*. *germanica* and *A*. *mellifera ligustica* both in terms of predator–prey coevolution [49] and in terms of potential defense strategies that can be used by native bees against alien species such as *V*. *velutina*.

## Supporting information

S1 Photo Predator and prey involved in a physical encounter. (PNG)

S2 Photo Formation of a ball of bees around a wasp until the latter is killed or becomes harmless (balling). (PNG)

S1 Movie Guard bees stop the intruder wasp at the entrance of the hive. (MOV)

S2 Movie Individual honey bee defense under wasp attack. (MOV)

S3 Movie Several individual bees help a nest mate involved in a conflict. (MOV)

S1 Data File Raw experimental data. (XLSX)
