# Supplementary figures and images for: Agonistic interactions between the honeybee (Apis mellifera ligustica) and the European wasp (Vespula germanica) reveal context-dependent defense strategies
Source: PLoS One. 2017 Jul 5;12(7):e0180278. doi: 10.1371/journal.pone.0180278 (PMC5497986; doi:10.1371/journal.pone.0180278)

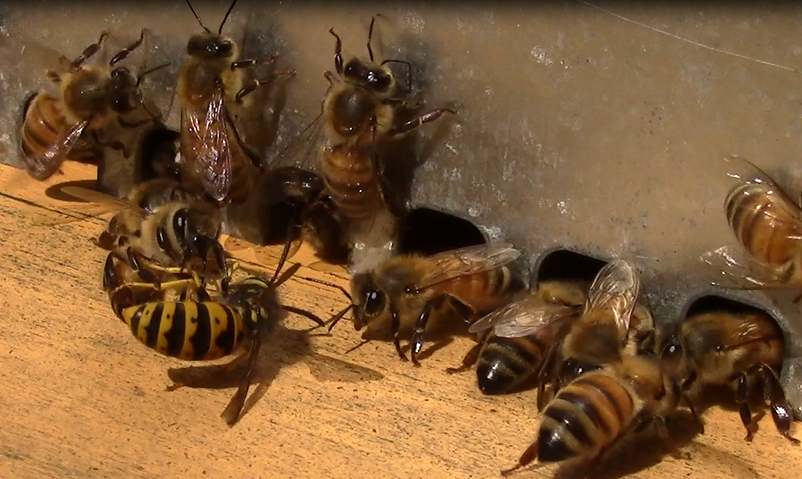

Supplement: S1 Photo — (PNG) [file pone.0180278.s001.png]

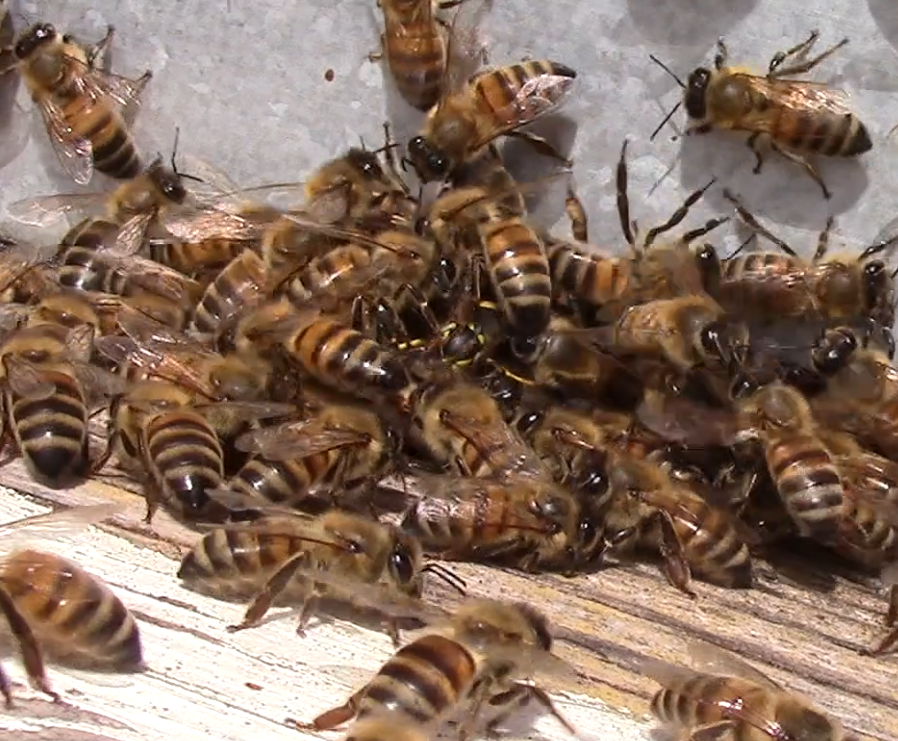

Supplement: S2 Photo — (PNG) [file pone.0180278.s002.PNG]
